# DNA Methylation of Human Choline Kinase Alpha Promoter-Associated CpG Islands in MCF-7 Cells

**DOI:** 10.3390/genes12060853

**Published:** 2021-06-01

**Authors:** Siti Aisyah Faten Mohamed Sa’dom, Sweta Raikundalia, Shaharum Shamsuddin, Wei Cun See Too, Ling Ling Few

**Affiliations:** School of Health Sciences, Health Campus, Universiti Sains Malaysia, Kubang Kerian 16150, Kelantan, Malaysia; sitiaisyahfatensadom@gmail.com (S.A.F.M.S.); sweta.raikundalia.jsk@gmail.com (S.R.); shaharum1@usm.my (S.S.); stweicun@usm.my (W.C.S.T.)

**Keywords:** DNA methylation, promoter, CpG island, *ckα*, cancer

## Abstract

Choline kinase (CK) is the enzyme catalyzing the first reaction in CDP-choline pathway for the biosynthesis of phosphatidylcholine. Higher expression of the α isozyme of CK has been implicated in carcinogenesis, and inhibition or downregulation of CKα (CHKA) is a promising anticancer approach. This study aimed to investigate the regulation of CKα expression by DNA methylation of the CpG islands found on the promoter of this gene in MCF-7 cells. Four CpG islands have been predicted in the 2000 bp promoter region of *ckα* (*chka*) gene. Six CpG island deletion mutants were constructed using PCR site-directed mutagenesis method and cloned into pGL4.10 vectors for promoter activity assays. Deletion of CpG4C region located between –225 and –56 significantly increased the promoter activity by 4-fold, indicating the presence of important repressive transcription factor binding site. The promoter activity of methylated full-length promoter was significantly lower than the methylated CpG4C deletion mutant by 16-fold. The results show that DNA methylation of CpG4C promotes the binding of the transcription factor that suppresses the promoter activity. Electrophoretic mobility shift assay analysis showed that cytosine methylation at MZF1 binding site in CpG4C increased the binding of putative MZF1 in nuclear extract. In conclusion, the results suggest that DNA methylation decreased the promoter activity by promoting the binding of putative MZF1 transcription factor at CpG4C region of the *ckα* gene promoter.

## 1. Introduction

Choline kinase (CK) is a cytosolic enzyme in the CDP-choline pathway that catalyzes the phosphorylation of choline to phosphocholine for the biosynthesis of phosphatidylcholine, the major phospholipid of eukaryotic cell membranes [1,2]. Human CK is encoded by two separate genes named *ckα* (*chka*) and *ckβ* (*chkb*)*. ckβ* codes for a single protein (CKβ), while *ckα* undergoes alternative splicing to produce CKα1 and CKα2 isoenzymes [3]. Increased activity of CKα isoform and higher levels of PCho have been implicated in human carcinogenesis. CK overexpression increases the invasiveness and drug resistance of breast cancer cells [4]. Abnormal expressions of *ckα* were also detected in various human cancers such as colorectal, lung, ovary, and prostate adenocarcinomas [5,6,7].

Over the years, enormous efforts have been focused on investigating the expressions of *ckα* in different cancer cells which led to the use of CK inhibitors as potential anticancer agents [8,9,10]. However, less attention has been given to the regulation of *ckα* gene expression, especially by epigenetic mechanism. C*kα* gene transcription was first reported to be regulated by hypoxia via the binding of HIF-1α transcription factor to the hypoxia response element-7 (HRE7) in the promoter [11]. Subsequently, the binding of C/EBPβ to *ckα* promoter during retinoic-acid-induced neuronal differentiation was shown to induce *ckα* gene transcription [12]. More recently, it was demonstrated that KDM2B binding to *ckα* promoter represses the expression of this gene [13]. Post-transcriptional regulation of *ckα* gene expression involving miRNAs has also been reported. Cancer cells transfected with miR-876-5p [14] and miR-367-3p [15] both downregulated the *ckα* mRNA levels and induced apoptosis. Epigenetic processes are natural and vital to many biological functions in an organism, and abnormal epigenetic changes often lead to dysregulation of developmental activities [16]. DNA methylation is the most well-studied epigenetic mechanism that is involved in diverse cellular functions, including the silencing of transposable elements, inactivation of viral sequences, maintenance of chromosomal integrity, X-chromosome inactivation, and transcriptional suppression of a large number of genes [17,18]. In somatic cells, DNA methylation occurs at cytosine in any context of the genome but predominantly in a cytosine-phosphate-guanine (CpG) dinucleotide context [19]. Methylated CpGs augment transcription repression by a number of processes, including the direct blockage of transcription initiation complexes from binding to DNA promoter regions and recruitment of transcriptional repressor complexes, including methyl CpG binding proteins (MBPs) that bind at methylated DNA sequences [20]. Aberrant methylation levels have been postulated to inactivate tumor suppressors and activate oncogenes, which lead to carcinogenesis [21].

In mammals, methylation occurs predominantly at the CpG dinucleotides, which are extremely depleted in the genome except at a short stretch genomic region termed as CpG islands, which are usually located at gene promoters [22]. DNA methylation of CpG islands especially on the promoter of a gene is one of the mechanisms that regulates the gene expression at transcriptional level. While the CpG dinucleotides in the genome are heavily methylated, the CpG dinucleotides in these islands remain unmethylated. Inactivation of numerous genes has been associated with the increased CpG island methylation in tumors such as *hMTLH1*, *p16*, *MGMT*, *BRCA1*, and *CCDN2* [23,24]. Hence, methylation of CpG islands is an important mechanism for gene inactivation in the prevention of tumor growth and development. Our preliminary work with HeLa cell line also showed that treatment with epigenetic drugs increased the methylation level of a specific CpG island in *ckα* gene promoter and affected the *ckα* promoter activity and gene expression level [25]. Thus, this study aimed to further investigate the effect of DNA methylation on the *ckα* gene promoter activity. The results suggest that the transcriptional control of *cka* gene involves DNA methylation of CpG islands located in the promoter region.

## 2. Materials and Methods

### 2.1. In Silico Analysis of ckα Promoter Region for CpG Islands and Transcription Factor Binding Sites

The CpG islands within 2000 bp upstream of *ckα* gene ATG start site (transcript NM_001277) were identified using MethPrimer (http://urogene.org/methprimer, accessed on 2 March 2017) and EMBOSS CpGPlot (https://www.ebi.ac.uk/emboss/cpgplot, accessed on 2 March 2017). EMBOSS CpGPlot defines CpG island as a region with observed/expected ratio > 0.60, length of > 200 bp, and GC content > 50%, while MethPrimer definition of CpG island is a region with observed/expected ratio > 0.60, length of > 100 bp, and GC content > 50%. The putative transcription factor binding sites were predicted using MatInspector 8.0 [26] and Alggen PROMO [27].

### 2.2. Cell Culture

The human breast adenocarcinoma cell line (MCF-7, ATCC^®^ HTB-22™) was cultured in high-glucose Dulbecco’s modified Eagle’s medium (DMEM) supplemented with 10% heat-inactivated fetal bovine serum (FBS) (Thermo Fisher Scientific, Waltham, MA, USA) and 100 µg/mL penicillin and streptomycin (Merck, Darmstadt, Germany). The human mammary epithelial cells (MCF10A, ATCC^®^ CRL-10317™) were cultured and maintained in DMEM/F12 (Nacalai Tesque, Kyoto, Japan), supplemented with 5% heat-inactivated horse serum (Thermo Fisher Scientific, Waltham, MA, USA), 20 ng/mL of epidermal growth factor (Nacalai Tesque, Kyoto, Japan), 0.5 µg/Ml of hydrocortisone (Nacalai Tesque, Kyoto, Japan), and 100 µg/mL penicillin and streptomycin (Merck, Darmstadt, Germany). Cells were maintained at 37 °C in a humidified atmosphere of 5% CO_2_ in a cell-culture incubator (SHEL LAB, Cornelius, OR, USA).

### 2.3. Treatment of Cells with 5-Azacytidine and Budesonide

Cells were treated with either DNA demethylating agent, 5-azacytidine (1 µM) (Abcam, Cambridge, UK), for 96 h [28] or DNA methylating agent, budesonide (70 µM), (Abcam, Cambridge, UK) for 24 h [29]. The culture medium with 5-azacytidine was replaced every 24 h with fresh medium containing the same concentration of 5-azacytidine. DMSO (Merck, Darmstadt, Germany) was added instead of the epigenetic drugs for negative controls.

### 2.4. Detection and Quantification of DNA Methylation Level

The detection and quantification of methylated DNA level was carried out using methylated-DNA IP kit (Zymo Research, Irvine, CA, USA) according to the manufacturer’s instructions. In brief, genomic DNA was sheared to 300–800 bp fragments using NEBNext^®^ dsDNA Fragmentase^®^ (New England Biolabs, Ipswich, MA, USA). Next, a mixture of 50 µL consisting of 300 ng of the fragmented DNA, DNA denaturing buffer, and 1 µL of 200 µM control DNA was incubated at 98 °C for 5 min to denature the double stranded DNA. The solution was transferred into a mixture containing 250 µL MIP buffer, 15 µL ZymoMag Protein A and 1.6 µL mouse anti-5-methylsytosine. The antibody/Protein A/DNA mixture was incubated at 37 °C for 1 h on a rotator before placing the tube on a rack with a magnetic rod to allow clustering of the beads. The antibody/Protein A/DNA complexes were washed three times with MIP Buffer. The immunoprecipitated DNA fragments were eluted with 15 µL DNA elution buffer and incubated at 75 °C for 5 min followed by centrifugation at 1800× *g* for 2 min. The recovered DNA was used for the subsequent DNA methylation experiment. The methylation levels of the enriched methylated genomic DNA were analyzed by conventional PCR using KOD Hot Start DNA Polymerase (Merck, Darmstadt, Germany) according to the manufacturer’s protocol. The primers used are shown in Table 1.

### 2.5. Construction of ckα Promoter-Luciferase Reporter Plasmids

The deletion of CpG island from the full-length *ckα* promoter was carried out using site-directed mutagenesis (SDM) by two-step PCR according to Ho et al. [30], in which the full-length pGL4.10-ckα(-2000/+9) plasmid was used as the DNA template. The PCR products were cloned into the *Nhe*I/*Hind*III digested pGL4.10 [*luc2*] vector (Promega, USA) to produce recombinant plasmid: namely, pGL4.10-ckα(∆CpG1), pGL4.10-ckα(∆CpG2), pGL4.10-ckα(∆CpG3), pGL4.10-ckα(∆CpG4A), pGL4.10-ckα(∆CpG4B), and pGL4.10-ckα(∆CpG4C). A pGL4.10-ckα-mut_MZF1 plasmid was constructed, in which mutations were introduced within the MZF1 transcription factor binding sites to determine the effects of bases substitution in *ckα* promoter activity. The primers used to introduce the mutations are listed in Table 1. All deletion constructs were verified by DNA sequencing.

### 2.6. Transfection and Luciferase Assay

*ckα* mutant promoter constructs were transiently transfected into MCF-7 cells using Lipofectamine^®^ 2000 (Thermo Fisher Scientific, Waltham, MA, USA) according to the manufacturer’s instruction. MCF-7 cell line was chosen based on our previous study that showed *ckα* promoter as an active promoter and having the highest promoter activity compared to the other cell lines [31]. Twenty-four hours prior to transfection, MCF-7 cells were seeded at a density of 1.0 × 10^5^ cells per well in a 96-well plate and grown in DMEM growth medium without antibiotics. The cells in each well were co-transfected with 200 ng of *ckα* promoter-luciferase constructs in pGL4.10[*luc2*] vector and 2.5 ng of internal control R*enilla* luciferase vector, pGL4.73[*hRluc*/*SV40*] (Promega, Madison, WI, USA), as the control for transfection efficiency. Twenty-four hours after transfection, cells were harvested and assayed using Dual-Glo^®^ luciferase assay system (Promega, Madison, WI, USA). The luciferase activities were measured by GloMax^®^ 20/20 Luminometer (Promega, Madison, WI, USA). Promoter activity was expressed as relative firefly luciferase activity after normalization to *Renilla* luciferase activity.

### 2.7. In Vitro Methylation of ckα Promoter-Reporter Plasmid

In vitro methylation of plasmid DNA was performed using CpG methyltransferase (M.SssI) (New England Biolabs, Ipswich, MA, USA). Plasmid DNA (1 µg) was treated with 1 unit of M.SSsI enzyme in a total volume of 20 µL in the presence of 1x M.SssI buffer and 160 µM *S*-adenosylmethionine (SAM). The mixture was incubated at 37 °C for 3 h, followed by heat inactivation at 65 °C for 20 min. Complete methylation was confirmed by digestion with the methylation-sensitive endonuclease *Hpa*II (New England Biolabs, Ipswich, MA, USA).

### 2.8. Electrophoretic Mobility Shift Assay (EMSA)

MCF-7 cell nuclear extracts were prepared using NE-PER nuclear and cytoplasmic extraction reagents (Thermo Fisher Scientific, Waltham, MA, USA) according to the manufacturer’s protocol. The biotinylated and unlabeled probes were synthesized by Integrated DNA Technologies (Coralville, IA, USA) and listed in Table 2. The complementary probes were mixed at 1:1 molar ratio by heating at 95 °C for 5 min followed by cooling down to the probe annealing temperature by 1 °C decreased per minute. The reactions were carried out in accordance with manufacturer’s protocol for Lightshift^®^ chemiluminescent EMSA kit (Thermo Fisher Scientific, Waltham, MA, USA). Each binding reaction consists of 1X binding buffer, 2.5% glycerol, 5 mM MgCl_2_, 50 ng/µL poly deoxyinosinic-deoxycytidylic (dI•dC), 0.05% NP-40, MCF-7 nuclear extract, 50–200 fmol of biotin-labeled target DNA, and distilled water in a total volume of 20 µL. The mixture was incubated on ice for 5 min before being added with the biotin-labeled DNA probes. DNA-protein complexes were electrophoresed on a 6% non-denaturing acrylamide gel in 0.5× tris-borate-EDTA (TBE) buffer. The biotin-labeled DNA was transferred onto a Biodyne B nylon membrane (Thermo Fisher Scientific, Waltham, MA, USA) and crosslinked by exposure to a UV light using UV transilluminator (Spectronics, USA) at 312 nm for 15 min. The signal was developed with the chemiluminescent nucleic acid detection module kit (Thermo Fisher Scientific, Waltham, MA, USA). The signal was detected by Fusion FX chemiluminesce imaging system (Vilber Lourmat, Collégien, France).

### 2.9. Statistical Analysis

Statistical analyses were performed using the Student’s *t*-test and one-way analysis of variance (ANOVA) with the Bonferroni post hoc test. GraphPad Prism version 6.0 was used to analyze the data, and the value of *p* < 0.05 was considered to be statistically significant. Data were presented as mean ± standard error of mean (SEM) from at least duplicates of two independent experiments.

## 3. Results

### 3.1. Construction of ckα Promoter-Luciferase Reporter Plasmids

**Figure 1:**
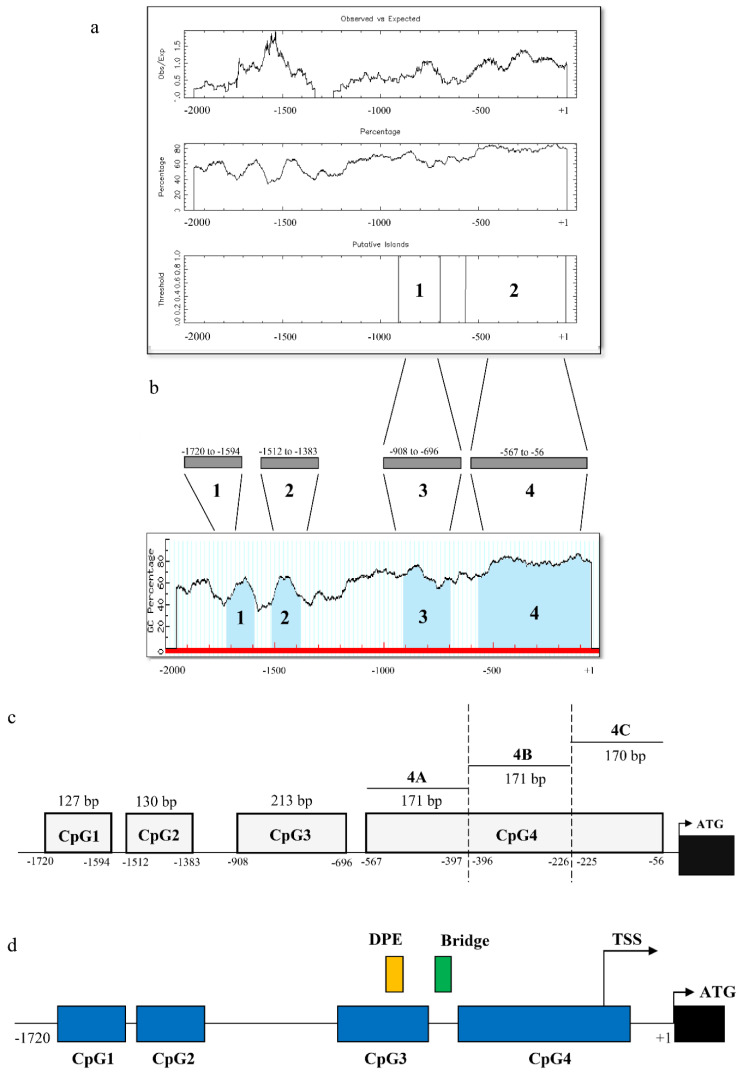
Identification of CpG islands of *ckα* promoter using: (**a**) MethPrimer and (**b**) EMBOSS CpGPlot; (**c**) CpG4 was divided into three smaller fragments which are CpG4A, CpG4B, and CpG4C and (**d**) the regulatory elements predicted in *cka* gene promoter. The blue boxes indicate the CpG islands. The yellow and green boxes indicate the downstream promoter element (DPE) and Bridge element, respectively. The transcription start site (TSS) was identified from DBTSS and ATG start codon are shown in bold uppercase letters. Black box indicates the first exon.

The MethPrimer analysis predicted four putative CpG islands within 2000 bp upstream from the ATG translation start site of *cka* promoter, while the EMBOSS CpGPlot predicted two CpG islands within the same region of *cka* promoter. Four CpG islands were located at regions between −1720 and −1594 (CpG1), −1512 and −1383 (CpG2), −908 to −696 (CpG3), and −567 to −56 (CpG4), when predicted using MethPrimer. The predicted location of the CpG islands using EMBOSS CpGPlot was consistent and overlapped with the analysis by MethPrimer at the third and fourth CpG islands. (Figure 1a). CpG4 was subsequently divided into three smaller fragments, namely CpG4A, CpG4B, and CpG4C (Figure 1c) for a more detailed characterization. Our analysis also showed that *cka* promoter lacks CAAT box and TATA box and contains two core promoter elements, namely the downstream promoter element (DPE) and Bridge element, which are a typical characteristic of GC-rich promoters (Figure 1d). The transcription start site in Figure 1d was identified from DataBase of Transcription Start Site (DBTSS) (https://dbtss.hgc.jp/, accessed on 1 August 2019) [32], it is located at chr11: 68,121,388 in the hg38 genome assembly.

### 3.2. Identification of Regulatory CpG Islands in the ckα Promoter

**Figure 2:**
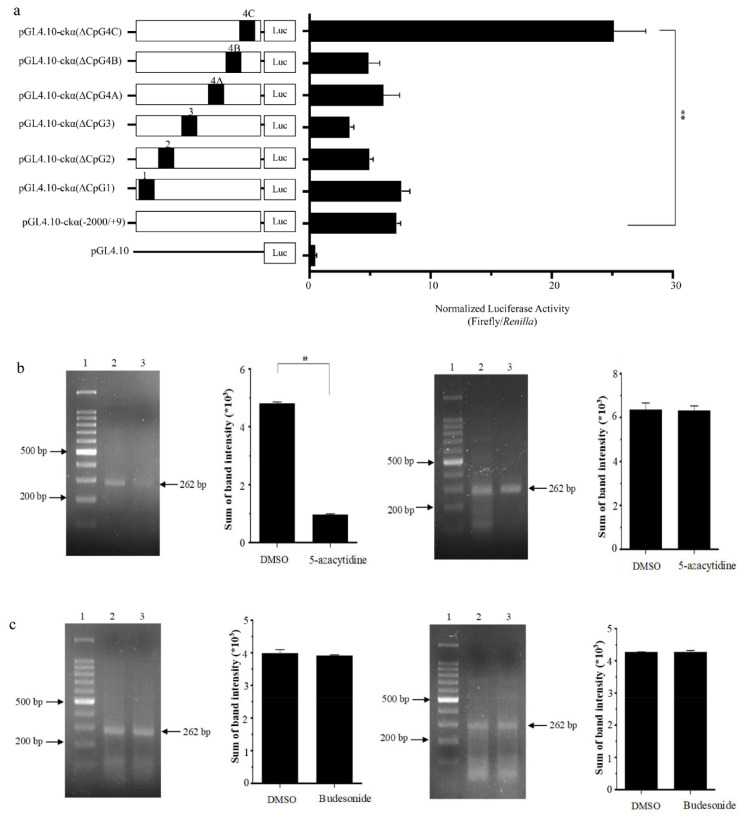
Deletion analysis of *ckα* CpG island and DNA methylation levels of CpG4C after epigenetic drug treatments. (**a**) Promoter activity of CpG island deletion constructs in MCF-7 cells. The closed rectangle on the left panel indicates the deletion of the CpG island. DNA methylation levels of CpG4C region after (**b**) 5-azacytidine and (**c**) budesonide treatments of MCF-7 (left panels) and MCF-10A (right panels) cells. Each bar represents the mean ± SEM of triplicate samples for three independent experiments. Statistical analysis was performed using Student’s *t*-test (** *p* < 0.01). Lane 1: size marker; 2: negative control; and 3: treated.

To elucidate the role of different CpG islands in *ckα* promoter activity, a total of six CpG island deletion constructs were made: namely, pGL4.10-ckα(∆CpG1), pGL4.10-ckα(∆CpG2), pGL4.10-ckα(∆CpG3), pGL4.10-ckα(∆CpG4A),pGL4.10-ckα(∆CpG4B), and pGL4.10-ckα(∆CpG4C). As shown in Figure 2a, the deletion of CpG4C resulted in a significant increase in promoter activity by ~4-fold (compared to the full-length promoter) and ~55-fold (compared to the promoter-less control). This indicates the presence of important negative regulatory elements within CpG4C. On the other hand, a ~2-fold reduction of promoter activity was observed when CpG3 was deleted indicating the presence of positive regulatory elements in this CpG island. The deletion of CpG1, CpG2, CpG4A, and CpG4B did not cause any significant changes of promoter activity compared to the full-length reporter construct. Due to its prominent effect on *ckα* promoter activity, CpG4C was selected for further investigation in subsequent experiments.

The effect of epigenetic drugs treatment specifically on the DNA methylation level of CpG4C region was determined in breast normal and cancer cells. As shown in Figure 2b,c, only the treatment of MCF-7 cells with 5-azacytidine resulted in significant decrease (~5-fold) of DNA methylation at CpG4C. MCF-7 cells treated with budesonide did not show any significant difference compared with negative control. No significant effect was observed for normal cells (MCF-10A) treated with both drugs. The results suggest that the CpG4C region is methylated in breast cancer cells, and 5-azacytidine could be used to modulate the methylation level at this CpG island.

### 3.3. Activity of In Vitro Methylated ckα Full-Length and CpG4C Deletion Promoter Constructs

**Figure 3:**
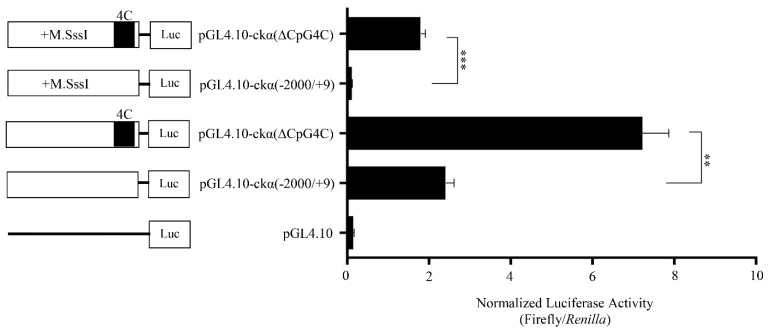
Effects of methylation by M.SssI on *ckα* promoter activity. Schematic structures of reporter construct are shown on the left. Each bar represents the mean ± SEM of triplicate samples for three independent experiments. Statistical analysis was performed using Student’s *t*-test (** *p* < 0.01; *** *p* < 0.001).

To further investigate the effect of DNA methylation on the promoter activity of full-length and CpG4C deletion constructs, the promoter constructs were methylated in vitro by CpG-specific methyltranferase enzyme, M.SssI. The promoter activity of methylated full-length promoter was significantly lower than the methylated CpG4C deletion mutant and unmethylated full-length promoters by ~16-fold and ~22-fold, respectively (Figure 3). The results suggest that CpG4C contains elements for the binding of a suppressor transcription factor and its binding is induced by DNA methylation.

### 3.4. Identification of the Regulatory Elements in the CpG4C of ckα Promoter

**Figure 4:**
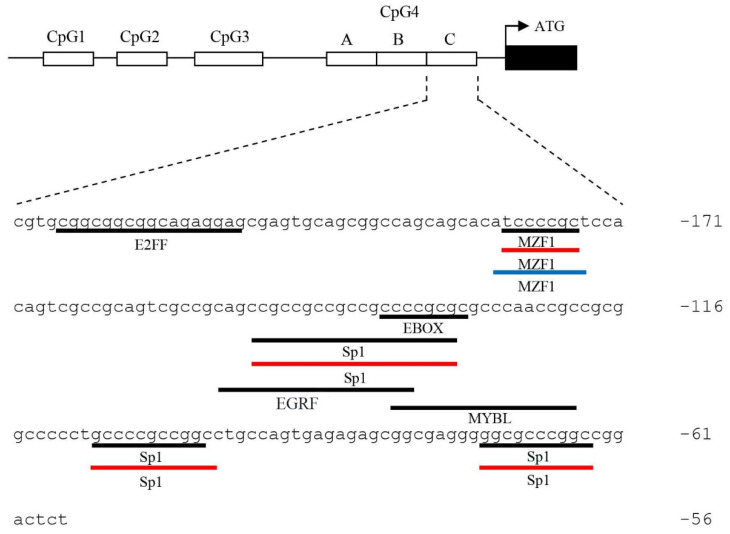
The transcription factor binding elements (underlined sequence) predicted within CpG island 4C of *ckα* promoter using MatInspector (black underline), Alggen PROMO (red underline) and DRAF omicX (blue underline). The numbers shown on the right panel indicate the number of bases from ATG translation start site.

Multiple consensus sequences recognized by transcription factors were predicted within CpG4C of *ckα* promoter using MatInspector, Alggen PROMO, and DRAF omicX (Figure 4). Four mutant constructs namely pGL4.10-ckα-mut_Sp(-73/-64), pGL4.10-ckα-mut_Sp(-108/-99), pGL4.10-ckα-mut_Ebox(-136/-127), and pGL4.10-ckα- mut_MZF-1(-181/-175) were constructed to disrupt the binding of the predicted Sp1, Ebox, and MZF-1 transcription factors to the CpG4C. The promoter activities of the mutant constructs were compared to those of the full-length and CpG4C deletion constructs.

**Figure 5:**
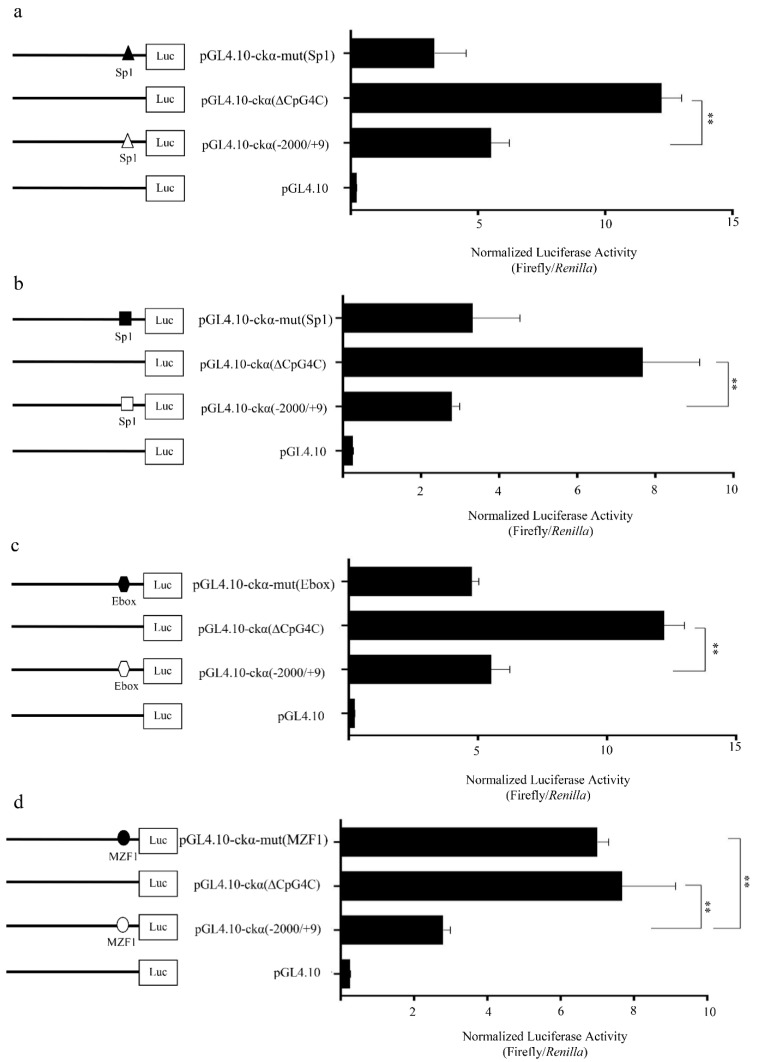
Effect of putative (**a**) Sp1(-73/-64); (**b**) Sp1(-108/-99); (**c**) Ebox(-136/-127); and (**d**) MZF1(-181/-175) binding site mutations on *ckα* promoter activity. Binding sites for the putative transcription factors are indicated with open and closed circles for wildtype and mutated sequence, respectively. Each bar represents the mean ± SEM of triplicate samples for three independent experiments. Statistical analysis was performed using Student’s *t*-test (** *p* < 0.01).

The mutated Sp1(-73/-64), Sp1(-108/-99), and Ebox(-136/-127) did not show significant changes in the promoter activity, suggesting that these three transcription factors were not involved in the suppression of *ckα* promoter activity (Figure 5a–c). Mutation of the putative MZF1 binding element on the other hand significantly increased the promoter activity to ~3-fold of the full-length promoter construct (Figure 5d). No significant difference was observed between pGL4.10-ckα-mut(MZF1) and pGL4.10-ckα(∆CpG4C), suggesting that the loss of MZF1 is the main reason that contributes to the higher pGL4.10-ckα(∆CpG4C) promoter activity. Overall, it can be postulated that MZF1 is a negative regulatory element in *ckα* promoter and DNA methylation at CpG4C promotes the binding of this transcription factor.

### 3.5. DNA Methylation Promotes the Binding of Putative MZF1 to ckα Promoter

**Figure 6:**
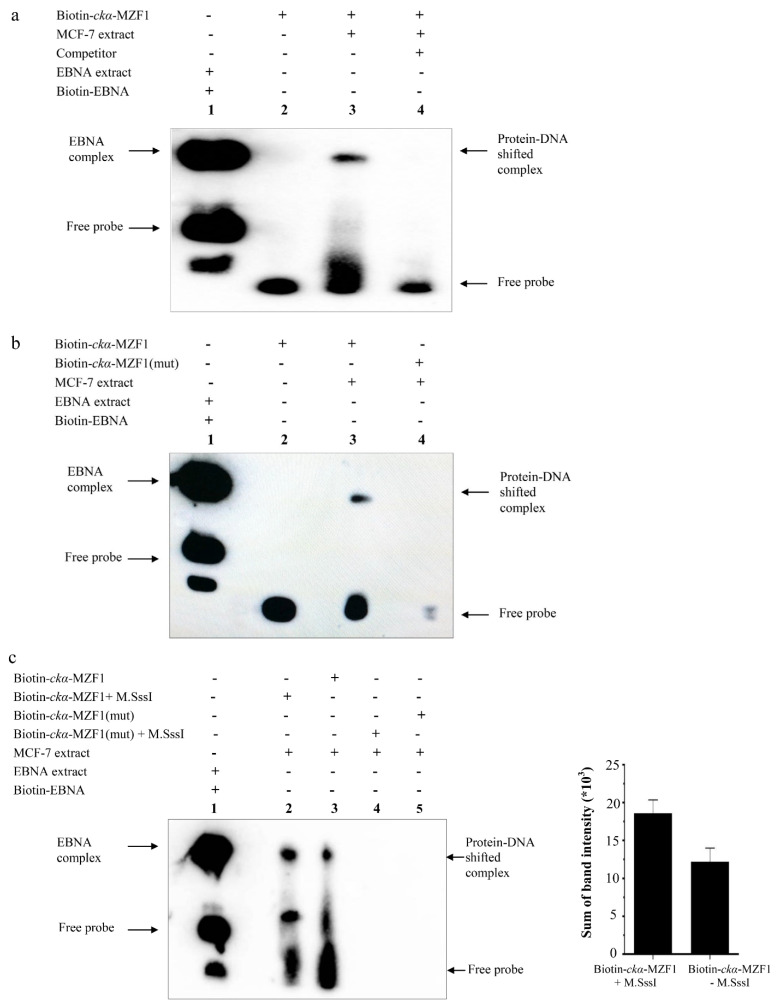
DNA methylation induces the binding of MZF1 to CpG4 of *ckα* promoter. (**a**) EMSA showing putative MZF1 transcription factor binding to *ckα* promoter; (**b**) mutation of MZF1 binding site abolishes the binding of putative MZF1 to *ckα* promoter; and (**c**) in vitro methylation CpG4 promotes the putative MZF1 binding. Histogram depicting band intensity data obtained from ImageJ. The error bars represent SEM from two independent experiments.

EMSA with a biotin-labeled DNA probes containing the MZF1 element in CpG4C was used to assess the binding of transcription factors in MCF-7 nuclear extract. Figure 6a shows a protein–DNA shifted complex, indicating the binding of a transcription factor to the MZF1 binding site. The binding specificity of putative MZF1 in the shifted complex was verified by competition binding assay and EMSA using mutated MZF1 binding sequence. No protein–DNA complex was formed when 100-fold molar excess of unlabeled competitor DNA was added (Figure 6a), and mutation of MZF1 binding site abolished the protein–DNA complex (Figure 6b). In vitro methylation of the probes by M.SssI increased the binding of putative MZF1 transcription factor as shown by higher band intensity of protein–DNA shifted complex compared to unmethylated probes (Figure 6c). The result suggests that DNA methylation promotes the binding of putative MZF1 transcription factor at CpG4C to downregulate *ckα* promoter activity.

## 4. Discussion

The consistent association of CpG islands with the promoter regions suggested its potential involvement in the transcriptional regulation of a gene. Approximately 60% of human genes are associated with CpG islands, which include all the housekeeping genes and about half of the tissue-specific genes [33,34]. In this study, CpG islands were identified in the 2000 bp promoter region upstream of *ckα* gene according to the criteria reported by Hackenberg et al. [35]. The absence of TATA-box and the presence of high GC-rich sequences in the *ckα* promoter are features commonly found in the family of housekeeping genes. Generally, human housekeeping gene promoters contain high GC content, high occurrence of CpG islands, and depleted TATA-box [36]. The TATA-less promoters in mammals are typically characterized by high prevalence of G-quadruplex-promoting sequences and CpG islands [37]. Since CpG islands are prone to DNA methylation, the presence of CpG islands in the *ckα* promoter suggested that DNA methylation could play a significant role in the transcriptional regulation of *ckα* gene.

In the current study, 5-azacytidine treatments significantly changed the methylation levels of CpG4C region in the MCF-7 breast cancer cells but not in the MCF-10A normal cells. On the other hand, budesonide did not show any significant effect on the methylation levels in both cell lines. The demethylating agent, 5-azacytidine, has been widely used as the DNA methylation inhibitor to induce gene expression. The covalent trapping of DNA methyltransferases (DNMTs) by 5-azacytidine depletes its activity, resulting in the demethylation of DNA. As the nucleoside analogue inhibitors of DNMTs, 5-azacytidine and 5-aza-deoxycytidine have been widely used in the attempts to reverse abnormal hypermethylation in cancer cells and exert their effects through re-expression of silenced genes [38,39]. Meanwhile, budesonide has been used as the DNA methyltransferase activator to increase the methylation of DNA and ultimately decrease the growth rate of tumors [40]. Budesonide decreased the size of lung tumors in mice by modulating DNA hypomethylation and the gene expressions of several tumor markers [41]. Previously, our results showed that 5-azacytidine and budesonide produced opposite effects on *ckα* full-length promoter activity and gene expression [25], which was an indicator of *ckα* promoter activity being modulated by DNA methylation in breast cancer cells.

CpG4C segment of the fourth CpG island was identified to be associated with the suppression of *ckα* promoter activity and the presence of a repressive element in that region that could be modulated by DNA methylation. Our results subsequently showed that methylation of CpG4C in MCF-7 cells correlates with a decrease in the *ckα* promoter activity by promoting the binding of repressive MZF1 transcription factor to this region of the CpG island. The suppression of gene expression by DNA methylation has been extensively documented by various studies [42]. In vitro studies have identified sequence-specific transcription factors that preferentially bind to the methylated CpGs (mCpGs) over unmethylated ones. Rishi et al. (2010) reported that mCpGs within the *CRE* motif enhance the binding of the C/EBPα transcription factor, a protein essential for activation of differentiation in various cell types [43]. Similar with the observation in this study, DNA methylation at *NRBP1* promoter region increased the binding of the transcription factor TFAP2A and led to the suppression of *NRBP1* expression [44].

The formation of specific protein–DNA complex in EMSA supports the functional role of MZF1 binding site in CpG4C for the repression of *ckα* promoter activity. The slight increase in protein–DNA complex formation in the methylated probes shows that DNA methylation promotes the binding of putative MZF1. It is known that cytosine methylation changes the DNA structure, which has the potential to negatively or positively influence the binding of transcription factors to DNA [45]. MZF1 functions as oncogene and involves in the invasion and metastasis of various solid tumors [46]. MZF1 is modified by phosphorylation and SUMOylation that influence its role as gene repressor or activator [47]. Previously, the MZF1 transcription factor was shown to negatively regulate the *CD34* and *c-myb* promoter activities in hematopoietic embryonic stem (ES) cells upon binding to the MZF1 binding site in the promoter regions of both genes [48,49].

Now that a specific regulatory CpG4C has been identified in the *ckα* promoter, more experiments focusing on this region should be performed in the future to further support the findings of this study. Bisulfite pyrosequencing could be carried out to determine the methylation sites along CpG4C. ChIP-Seq and supershift experiments using different cell lines with a methylated and nonmethylated *ckα* promoter could be performed in the future to confirm the binding of MZF1 to CpG4C. In this study, not all the predicted transcription factor binding sites in CpG4C were mutated to investigate their roles in regulating *ckα* promoter activity. Studying all the predicted *cis*-acting elements might reveal the functions of other transcription factors in relation to DNA methylation. The effects of 5-azacytidine and budesonide treatments on *ckα* promoter activity and the binding of MZF1 observed in this study might be “indirect” due to the global changes in methylation pattern of the genome introduced by these drugs. The concentrations of MZF1, CpG binding proteins or chromatin-associated proteins that might affect or compete with the binding of MZF1 to CpG4C could change after the treatments. To address this concern, targeted methylation, or demethylation of specific sites in *ckα* promoter, such as the MZF1 binding site, by CRISPR-based approach could be attempted in the future. The engineered nuclease-deficient version of Cas9 (dCas) fused to the catalytic domain of DNA methyltransferase or Tet dioxygenase has been used to manipulate the methylation levels of specific genomic loci including the CpG islands in promoter regions [50,51].

## 5. Conclusions

In conclusion, the methylation of CpG islands at the promoter is a significant regulatory mechanism in controlling the transcription of *ckα* gene. This study shows that DNA methylation could elicit transcriptional repression of *ckα* gene by promoting the binding of putative MZF1 transcription factor to the CpG4C region of *ckα* promoter.

## Figures and Tables

**Table 1:** Primers used for methylated-DNA immunoprecipitation and PCR-based site-directed mutagenesis.

Name	Sequence 5′ to 3′
**Methylated-DNA IP (MeDIP)**	
ckα-CpG1-5′	TATCCTTAAATAAGACCATTTTGCC
ckα-CpG1-3′	TAGTAGAGACGGGGTTTCAT
ckα-CpG2-5′	AAAATTAGCCAGGCGTCGTG
ckα-CpG2-3′	GAGTTCACAGTCTTCCAGAAGCAA
ckα-CpG3-5′	CGAGCATCCTCAGTACCACGGA
ckα-CpG3-3′	AGCAGCCTCCTCCTGGGGCTCA
ckα-CpG4A-5′	TCCGAGGGGTCCAAGGAAAC
ckα-CpG4A-3′	TGAGCGGGGCCTGGCCGAA
ckα-CpG4B-5′	CCCCTCGACGCCCCGCCCCCTT
ckα-CpG4B-3′	TCGCTCCTCTGCCGCCGCCGCACG
ckα-CpG4C-5′	AGCGCGAGGGCGGGCTGTGAC
ckα-CpG4C-3′	TGCCCGACAGGCGGCCGAGGA
**CpG island deletion**	
ckα-∆CpG1-5′	TAAATAAGACCATTTTGCGTGGAGGCTAACACGATGAAACC
ckα-∆CpG1-3′	GGTTTCATCGTGTTAGCCTCCACGCAAAATGGTCTTATTTA
ckα-∆CpG2-5′	AATTAGCCAGGCGTCGTGCTCAAAAAAAAAACCAAAA AACATTTTTGC
ckα-∆CpG2-3′	GCAAAAATGTTTTTTGGTTTTTTTTTTGAGCACGACGCCT GGCTAATT
ckα-∆CpG3-5′	CTCAGTACCACGGGAGCCCCAGGAGG
ckα-∆CpG3-3′	CCTCCTGGGGCCTCCCGTGGTACTGAG
ckα-∆CpG4A-5′	GGGTCCAAGGAAACTTCGCCCAGGCCCC
ckα-∆CpG4A-3′	GGGGCCTGGGCGAAGTTTCCTTGGACCC
ckα-∆CpG4B-5′	CCCCGCCCCCCGTGCGGCGG
ckα-∆CpG4B-3′	CCGCCGCACGGGGGGCGGGG
ckα-∆CpG4C-5′	GGCCGGCGCTCCTGAGCCTAGTCCTC
ckα-∆CpG4C-3′	GAGGACTAGGCTCAGGAGCGCCGGCC
**Mutation at transcription factor binding site**	
ckα-mut(MZF1)-5′	CCCCCTTTCACGCCGGCCTGCCAGTGA
ckα-mut(MZF1)-3′	CGGCGTGAAAGGGGGCCGCGGCGGTT

**Table 2:** List of DNA probes used in EMSA. The MZF1 core sequence is underlined.

Names	Sequences
Biotin-*ckα*-MZF1	5′-CAGCAGCACATCCCCGCTCCACAGTCGCC-3′-Biotin
Biotin-*ckα*-mut(MZF1)	5′-CAGCAGCACAT*ta*C*t*GCTCCACAGTCGCC-3′-Biotin
*ckα*-MZF1 complementary probe	5′-GTCGTCGTGTAGGGGCGAGGTGTCAGCGG-3′
*ckα*-mut(MZF1) complementary probe	5′-GTCGTCGTGTAATGACGAGGTGTCAGCGG-3′
